# Musicians as “Makers in Society”: A Conceptual Foundation for Contemporary Professional Higher Music Education

**DOI:** 10.3389/fpsyg.2021.713648

**Published:** 2021-08-03

**Authors:** Helena Gaunt, Celia Duffy, Ana Coric, Isabel R. González Delgado, Linda Messas, Oleksandr Pryimenko, Henrik Sveidahl

**Affiliations:** ^1^Royal Welsh College of Music & Drama, Cardiff, United Kingdom; ^2^Royal Conservatoire of Scotland, Glasgow, United Kingdom; ^3^Academy of Music, University of Zagreb, Zagreb, Croatia; ^4^Escola Superior de Música de Catalunya, Barcelona, Spain; ^5^Association Européenne des Conservatoires, Académies de Musique et Musikhochschulen, Brussels, Belgium; ^6^Department of Social and Humanitarian Disciplines, Kharkiv National Kotlyarevsky University of Arts, Kharkiv, Ukraine; ^7^Rhythmic Music Conservatory, Copenhagen, Denmark

**Keywords:** musicking, cultural entrepreneurship, professional identity, professionalism, critical reflection, civic mission, higher music education, artistic citizenship

## Abstract

This paper considers the purpose, values and principles underpinning higher music education (HME) as one of the performing arts in a context of turbulent global change. Recognising complex challenges and opportunities in this field, HME is addressed from dual perspectives: educating the next generations of professional musicians, and higher music institutions’ (HMEIs) engagement in society. The paper has a particular focus on the sector within HME that is dedicated to intensive practical craft training for performers, composers, programmers, producers, managers, and teachers. We argue that there is an urgent need for fresh orientating frameworks through which to navigate HME’s development. We examine concepts such as artistic citizenship, social responsibility and civic mission increasingly perceived to be relevant to the sector, and we explore their connexions to concepts of artistic excellence, imagination and creativity, and musical heritage. We identify apparent dichotomies of value within contemporary HME, including between intrinsic and instrumental purpose in the arts, cultural heritage, and new work, artistic imagination and entrepreneurship, and we argue that creative tensions between what have hitherto easily been perceived as opposing concepts or competing priorities need to be embraced. To support our argument we draw on the particular ethnomusicological concept of “musicking,” and we look toward a partnering of artistic and social values in order to enable HME to respond dynamically to societal need, and to continue to engage with the depth and integrity of established musical traditions and their craft. Based on this discussion we propose a conceptual foundation: the “musician as a maker in society,” in which developing vision as a musician in society, underpinned on the one hand by immersion in musical artistry and on the other hand sustained practical experience of connecting and engaging with communities, offers invaluable preparation for and transition into professional life. We propose that this idea, connecting societal and artistic vision and practise, is equally essential for HMEIs as it is for musicians, and sits at the heart of the roles they evolve within their local communities and wider society.

## Shifting Ground for Higher Music Education

Higher music education (HME) holds a leading position within music education as a whole, providing a pipeline into an array of professional music fields and curating research and innovation agendas. Higher music education institutions (HMEIs) particularly offering practical disciplines in music performance, composition and creative practise form the focus of this paper. They usually engage a strong international student body, with a large proportion of teaching staff also working as professional music practitioners. In some contexts such institutions are referred to as “conservatoires,” “music academies,” or form part of “universities of the arts.” For the purpose of this paper, however, the term HMEI is used in order to be inclusive across this nomenclature. Nevertheless, it is not intended for this to suggest that the content of the paper reflects the full range of HME delivered across universities.

Many specialist HMEIs of the kind described in this paper have a focus on western classical music. Some also offer jazz, popular, and indigenous folk musics—in Europe, the International Academy of Music and Performing Arts Vienna, and the Sibelius Academy, University of the Arts Helsinki provide longstanding examples. A smaller but growing group of institutions, such as the Irish World Academy of Music and Dance (Limerick) or the Institute of Contemporary Music Performance (London), does not address western classical music at all. Questions of artistic quality, profile, and global reach tend to be central concerns for all these HMEIs, often reflected in their mission and vision statements (Jørgensen, 2017) and in ways that international prizes and the profiles of the most successful “star” alumni and staff are promoted. Furthermore, such HMEIs have established a prominent position in the ecology of HME, often perceived to be guardians of artistic performance values and standards of musical excellence (Tregear et al., 2016). They have also been on a significant journey over several decades to evolve quality assurance and enhancement frameworks (Jørgensen, 2009; European Association of Conservatoires, 2010).

With many musicians and music organisations confronting rapidly shifting professional landscapes (Tolmie, 2020), HMEIs have over the last 30 years increasingly been adapting elements of their programmes, recognising the need to support portfolio careers and to enhance employability (Gembris and Langner, 2005; Bennett and Hannan, 2008; Bennett, 2016; Munnelly, 2020). Initiatives have focused, for example, on education in creative and cultural entrepreneurship (European Association of Conservatoires, 2014; Amussen et al., 2016; Renew, 2019); decolonising curriculum, including greater diversity in composers, performers and teachers represented in performance, as well as more diverse musics and musical practises being studied (Myers, 2016; Avis, 2019; European Association of Conservatoires, 2020); promoting intercultural collaboration and learning through international exchange and partnership (Grant, 2018; Bartleet et al., 2020); and exploring digital technologies in creating content (Ruthmann and Hebert, 2012), engaging audiences (Tsiouslakis and Hytönen-Ng, 2016; Toelle and Sloboda, 2019), and opening up access to learning (Krebs, 2017; Merrick, 2018). Some institutions have begun to promote forms of artistic citizenship in their graduate outcomes, and to explore how to combine ongoing practical craft training with addressing major societal changes including both social and environmental issues (Sarath et al., 2014; Tregear et al., 2016; Grant, 2018; Angelo et al., 2019; Westerlund and Gaunt, 2021).

Institutionally HMEIs are also beginning to engage with a broader civic mission, responding to fundamental questions about the public service of these institutions (Tregear et al., 2016), about how different groups in society are empowered to engage in music (Renshaw, 2013) and indeed who is enabled to train as a professional musician. Such initiatives align with similar expansion in the mission of higher education more broadly, deepening attention to civic mission, greater local community engagement and concern for societal impact, entrepreneurialism, and ecological development alongside commitments to research and specific discipline teaching (Barnett, 2011, 2012).

These trends in higher education have been accompanied by significant developments in curriculum design, modes of study, and pedagogies to support learning for contemporary and future contexts (Boud and Molloy, 2012). Constructivist paradigms of learning and teaching are now widely accepted, and are being used flexibly across a range of “engaged” environments such as work placements and internships, and projects connecting with diverse societal groups to create a more “connected” experience (Fung, 2017). Within HMEIs, a growing focus on pedagogical practises has also emerged (Gaunt and Westerlund, 2013; Hanken, 2016; Rowley et al., 2019). Where institutions have strong roots in western classical music, pedagogies have often been based on a tradition of apprenticeship, characterised by master practitioners passing on their skills and knowledge to the next generations through intensive contact (often on a one-to-one basis) and work-based learning environments (Kingsbury, 1988; Jørgensen, 2009). In many ways this represents an approach intimately connected to the professional field, and in recent years research has developed to unpack its distinctive qualities and consider its evolution within contemporary constructivist paradigms (López-Íñiguez et al., 2014; Gaunt, 2017; Carey et al., 2018; Coutts, 2019; Burwell, 2020). Alongside this, collaborative, enquiry-based and informal learning environments have gained prominence, complementing 1-2-1 apprenticeship (Gaunt and Westerlund, 2013; Born and Devine, 2015; Gies and Saetre, 2019); collaboration between different musical traditions and their diverse curriculum approaches and pedagogies has started to be embraced more fully (Schippers, 2010; Minors et al., 2017); and the demands and opportunities of expanding professionalism in the field of music and its implications for HME have begun to be assessed (Westerlund and Gaunt, 2021).

A further transformative trend has been the strengthening of student voice both within higher education generally and specifically in HME (European Association of Conservatoires, 2017; Coutts, 2018). As clear inheritors of the future of the professional music industries, and often arriving in HME with significant prior achievement and experience, students as partners have a critical role for HME in adapting to contemporary contexts, including for example in making a turn toward engaging with societal change and diverse communities.

Making change, however, has not necessarily been easy for HMEIs. With many contemporary contexts characterised by bewildering complexity, and some arts organisations as well as HMEIs facing a battle for survival alongside growing well-being pressures, which directions to pursue have not been self-evident. These challenges have been evidenced by the work, for example, of recent projects based in Europe that have turned explicitly to exploring societal change and its implications for evolving musical practises and professional education in HME: *ArtsEqual: The Arts as Public Service—Strategic Steps Toward Equality*^1^, a multi-disciplinary research project coordinated by the University of the Arts, Helsinki and funded by the Academy of Finland, and *Strengthening Music in Society*^2^, a development project of the European Association of Conservatoires (AEC) funded by Creative Europe. Both projects outline bold agendas, including participation in musical practises and access to HME. Each highlights the need for systemic perspectives, in order to make sense of contemporary complexities and enable appropriate change.

Within this situation, both the Covid-19 pandemic and the Black Lives Movement (BLM) sent seismic shockwaves through HME, stimulating developments unprecedented in their speed. Profound inequalities and their structural embedding within HMEIs, as in many organisations, were exposed, and initiatives to de-colonise curriculum, restructure admissions processes and staff recruitment gained momentum. The imperative to embrace digital technologies also become obvious. HMEI teachers who previously would have seen a video-conferenced lesson as certainly second best, become expert users almost overnight. LoLa (low latency) systems which allow musicians to play together without a noticeable time lag, became more widely used. This gave many institutions an unprecedented sense of how fast they could adapt in certain ways when they had to. Experiences of the pandemic and BLM also, however, intensified questions about how to engage with what can easily be experienced as competing or indeed conflicting priorities. The issues are not straightforward in working, for example, with the different priorities of a global industry and the importance of local engagement; or between agendas of equality, diversity and inclusion and existing institutional priorities, structures, and cultures (including commitments to “quality”); between digital and face-to-face environments; between sharing music-making informally in tiny settings and the experience of large-scale music venues; between free content and musicians’ need to be remunerated. As a consequence, institutions are having to return to critical issues about vision and purpose, to how they understand the relevance and value of music in societies, and to a range of associated moral and ethical questions inextricably involved in artistic practises.

### A Time to Strengthen Music in Society?

In revisiting purpose and vision for HME, questions cannot therefore be ignored about the ways in which musical practises are indeed of value in societies and the degrees to which these are realised, the roles musical practises may play within rapidly changing situations, and how they may be part of nurturing flourishing and inclusive societies for the long term. In many ways a contemporary zeitgeist is crying out for the creativity and humanity of music and the arts: their unique potential to uplift, heal, and engage people in expressing themselves, to help make sense of experience and challenge perspectives, and to contribute to building and sustaining communities (DeNora, 2000; Crossick and Kaszynska, 2016; Turino, 2016; Bazalgette, 2017; Pairon, 2019). Although live performance went through unprecedented restriction during the Covid-19 pandemic, it was also clear that the outpouring of remote, streamed performances as well as opportunities to participate in music making online reflected music’s resonance, for many at deeply personal levels. Wider policy on education and economic development has been transitioning from heightened attention to STEM (Science, Technology, Engineering, Mathematics) subjects to greater focus on a STEA(rts)M movement (Boy, 2013). Although this represents a long journey, policy continues to offer positive directions in some contexts (see for example European Commission, 2017). Equally the contribution of the creative industries to GDP is increasingly understood, calculated in the EU to be in the region of 4.5% of GDP and with a growth rate of 10% annually (Dronyuk et al., 2019). Changing forms of production and consumption have driven new music business models for the creative sector, and indeed dramatic shifts in the recording industry have made the field less centred on major labels (Negus, 2019) and have contributed significantly to diversifying markers of professional success. Rapid technological change has opened up new ways to create, learn and experience music, with network connectivity deeply affecting the ways in which we interact socially and musically (Waldron, 2018). Remixing tracks and social engagement with music via online platforms (Ruthmann and Hebert, 2012), or informal online instruction for learning instruments (Merrick, 2018), or experiencing music via VR systems that put the listener at the centre of an orchestra are commonplace. The generative possibilities of music making (in both familiar and novel ensemble formations) to help shape collective identities is gaining currency (Shelamay, 2011). EU policy (European Commission, 2017) has highlighted the contribution of the arts to wider social challenges, noting how they have engaged directly and indirectly with issues of inequality, migration, climate and environmental change, social justice, conflict and violence, loneliness, and isolation. It comes as no surprise then that the agenda for the latest Erasmus programme for HE is focused in line with the United Nations 2030 goals for sustainable development, highlighting diversity, and inclusion (European Commission, 2021). Musical practises may indeed be able to resonate with and connect people, make meaning, and contribute to shaping social relations and societal development (Hallam, 2010; Turino, 2016; Westvall and Aragão, 2019).

Nevertheless, music cannot be assumed to be a universal good, far from it (Taylor, 2016). musicians’ use for political propaganda, not least in contexts of profound conflict, and the ways in which mass gatherings for music have on occasion been targeted by terrorists counter unequivocally positive perspectives. In addition, some music projects with explicit social objectives such as the much-lauded El Sistema initiative originating in Venezuela, have been critiqued for inappropriate exercise of power and social control (Baker, 2014). Social dimensions of music-making raise multiple ethical and political issues (see for example Citron, 2000) that clearly need to be addressed as matters of urgency alongside aesthetic and imaginative concerns. While there is much potential for music to make a difference in diverse societies, achieving this calls for careful evolution of professional practises and professionalism in music (Westerlund and Gaunt, 2021), to find a fulcrum that enables human and societal concerns to be in reciprocal exchange with artistic values.

A social, moral and political core to music making may not, however, always sit comfortably with the directions that music has taken as a global industry. Perceptions of excellence have easily tended to focus on attributions of artistic skills and creativity distanced from local or societal orientation. In western classical music, for example, the ideal of the virtuoso soloist as the pinnacle of achievement has long been prominent (Kingsbury, 1988), accompanied by growing craft specialisation and technical standards. The last century has also seen an exponential rise in an international “star” culture (Marshall, 2013; Cook, 2018), with considerable economic value being derived from it. This has left many musicians who work to social and cultural objectives having lesser status (Rimmer, 2018). Tensions between the priorities of global star performance and locally-oriented practises are evident. Furthermore, public venues are being challenged over accessibility, elitism and cultural reproduction of economic, race and gender inequalities within their practises (Bull, 2019). With some large concert halls increasingly hard to fill and struggling to serve the diversity of their communities (Bradley, 2017), calls to widen access and evolve more inclusive practises (Johnson, 2002; National Endowment for the Arts, 2015) are set to grow. However, the Covid-19 pandemic has further highlighted enormous complexities around the role of professional musicians, how their work is valued and indeed may be remunerated in radically altered situations where the music industry will never be quite the same again, and relationships between live face-to-face interaction and digital work have shifted (World Economic Forum, 2020).

### Embracing Complexity to Envision HME

All in all there are systemic questions for HMEIs to address that reach potent and at times unspoken assumptions about who musical performance is for, how diverse people across cultures and with different levels of musical experience may engage and participate, and what roles professional musicians play in contemporary societies. From these follow more specific questions, including:

- In what ways may/must musicianship and artistic craft skills interact with societal awareness, social purpose and engagement?
- What foundations for lifelong employability do musicians need in the twenty-first century? What mix of vision, musical craft, fluency with digital innovations and research, social and cultural entrepreneurship, reflexivity and activism may be relevant?
- What roles may HMEIs develop within their communities? How may local attention combine with national and international engagement?
- In what ways may HMEIs address issues of systemic exclusion to embrace agendas of equality, diversity and inclusion?

In turning to these questions, we recognise the imperative critically to examine the conceptual foundations of HME. In the next section, we therefore consider relationships between artistic and social purpose in more detail. We draw out ways in which these have tended to be valued hierarchically and at times understood as a matter of conflicting polarities. We seek to re-map them dialogically to promote a “partnering of values” (Schmidt Campbell, 2016). Our aim is to deconstruct entrenched hierarchical relationships between artistic and social purpose, to explore their mutual dependence and the potential of negotiating creative tensions between them according to context. For HME, we look toward combining engagement with the depth and integrity of artistic traditions with the ability to respond dynamically to rapidly changing needs in societies: “Preserving and nurturing intrinsic value and partnering the intrinsic with the instrumental are legacies of this era” (Campbell, 2016, p. 46). Establishing a conceptual landscape for HME based on dynamic relationships that promote such a partnering of values, we suggest, offers a more accessible basis from which to ground reflection, reflexivity, and constructive decision-making in contemporary times.

## Moving Forward: From Competing Priorities to “Partnering Values”

### Artistry *and* Citizenship

*There should be no dividing line between artistic excellence and social consciousness* – Polisi (2016)

*After over a decade of being in the industry, I really recognise my position that people are watching me……Now my mission is different, I have a responsibility to this whole world* – Lady Gaga (2020)

Both Lady Gaga, one of the twenty-first century’s best-selling artists also known for her social activism and Joseph Polisi, cultural thinker and former President of the Juilliard School affirm the indivisibility of artistry and social awareness. Yet beyond the clear intention underlying these sentiments sits long-standing debate between the notion of “art for art’s sake” on the one hand, that looks exclusively through a lens of artistic coherence and value, and art for social purpose on the other hand, that looks through a lens of impact on people without necessarily embracing aesthetic concerns (see for example Koopman, 1998; Kleppe, 2016). With policy across numerous jurisdictions increasingly focusing on the socio-economic impacts of the arts, a plethora of research has emerged evidencing the instrumental value of music, and its contribution as one of the arts to building confidence and enhancing learning in children, to health and well-being in an ageing population and so on (Hallam, 2010; Smilde et al., 2014; Crossick and Kaszynska, 2016; Fancourt and Finn, 2019). Some of this work, however, has appeared to pay relatively little attention to artistic quality and artists’ creative concerns, and sits in striking contrast to the ways in which many HMEIs promote their work and celebrate success in terms of “artistic excellence,” competition wins, and recording contracts. It is also the case that there are plenty of musicians whose primary focus is artistically rooted in practical craft and employability more than in the nature and potential of socially-engaged performance.

A first contemporary dilemma then for HME concerns how to work productively with this debate and a continuum from art for art’s sake to art for social purpose, and to examine how artistic motivations may interact with societal awareness and social engagement. Multiple perspectives may be possible. In recent years the concept of “artistic citizenship” has come to the fore, seemingly bridging the divide. Elliott et al. (2016) critique the notion of the value of art lying primarily in aesthetic appreciation of the object (the master work, the musical score) rather than in “doing” music as a social practise. Examining opposition between artistry and citizenship, they assert that:

The arts are made by and for people, living in real worlds involving conflicts large and small. As such, the arts are also and invariably embodiments of people’s political and ideological beliefs, understandings, and values, both personal and collective (Elliott et al., 2016, p. 5).

This counters a nineteenth century view of artists as “inner-directed free spirits whose vision and work must not be contaminated by considerations ‘extrinsic’ to the formal or expressive qualities deemed resident in the artwork itself” (Elliott et al., 2016, p. 8), and it lines up with Polisi’s contention that the traditional “self-absorbed *artist*” is the wrong model for the *arts* in America in the twenty-first century.

Elliot, Silverman et al.’s conception of artistic citizenship, however, is uncompromising. Dominant notions of “responsibilities to each other,” “social-civic responsibilities,” and of a “conscientious artistic citizen” suggest that an artist’s responsibility in society can perhaps only be enacted properly in socially-inclusive participatory practises with a heavy lean toward instrumentalism, and limited attention to artistic concerns. The last 20 years have, however, seen plenty of objections to instrumentalism from the arts professions, and affirmation of artistic drivers underpinning “quality,” including within participatory contexts:

Basically, creative engagement in social settings becomes a pale shadow of what is possible if it is not driven by an artistic voice… (Renshaw, 2013, p. 46)

For Renshaw there is no question of diluting the artistic quality of practise and performance in socially-engaged contexts, although achieving this in practise may be demanding.

A different perspective is evident, for example, in a long tradition of successful artists “giving back” to the community after their career has peaked, and for instance opening music schools in impoverished communities, inspired by the El Sistema model developed in Venezuela (Baker, 2014). Gilberto Gil, musician and a former minister of Culture of Brazil, has articulated this perspective but is not dogmatic about it, and when asked whether every artist should always give something back to society, has commented:

That’s already there. By doing music, by singing, by performing, by addressing people, by communicating, by getting messages across, they are already doing that. So this kind of social responsibility, by committing in terms of social projects, this is not absolutely necessary. I think that art and cultural manifestation plays a role in itself as a public service. Just by being there. By communicating (Cobo, 2006, p. 21)

This lens on social engagement is much less prescriptive than that offered by Elliot et al. and proposes a connected model of the artist who carries out a public service “just by being there.”

Yet another perspective comes through the concept of activism, which as set out by Kuntz (2015), brings together imaginative, artistic and socially-oriented dimensions of practise. Hess (2019) elaborates on this further in the context of music, specifically within pre-HE learning and teaching:

Activism and music are enmeshed and inextricably connected. Music education and activism integrate similarly. Music learning intrinsically involves exploring social, political, historical, and cultural contexts, while musical activism can provide a significant mechanism for music learning. How might youth interact with music in music education in ways that validate their experiences and help them to develop their own unique voices? How might such interaction with music education contribute to social change? (Hess, 2019, p. 4)

Activism, like artistic citizenship, provides a praxis-oriented stance. It explicitly refers to imagination creating action for social change, and contrasts with Gil’s “existing as a performer and communicating the music” being a sufficient form of social engagement.

In any event, these different perspectives make it clear that there are diverse ways for musicians and HME alike to work with principles of “social engagement,” “activism,” or “artistic citizenship,” and to position themselves within the continuum of art for art’s sake through to art for social purpose. Furthermore, positions will evolve over time and across contexts, often in subtle and complex ways. Nevertheless, the spectrum of perspectives, and the nature of the continuum itself as a dynamic set of possibilities, cannot be taken for granted. It must be debated artistically, ethically and politically.

### “Musicking”

Critical reflection on this continuum and multiple issues within it is essential for contemporary HME. In order to support this, the ground-breaking ethnomusicological concept of “musicking” (Small, 1998), while not new, offers a helpful entry point. “Musicking” fundamentally stresses music as an active and interactional phenomenon, which immediately resonates with the practical orientation of HME discussed in this paper. Furthermore, “musicking” stresses music being situated in society rather than an abstract ideal. A distinction is made between music as *artefact* and music as *participation*:

To musick is to take part, in any capacity, in a musical performance, whether by performing, by listening, by rehearsing or practicing, by providing material for performance (what is called composition), or by dancing (Small, 1998, p. 9)

This is contrasted with a trend Small identifies in western societies of valuing the artefacts of music (the scores, recordings etc.) over the acts of creating, listening and responding to music.

“Musicking” refocuses attention to the social, interactive, and actively participatory nature of music-making in diverse contexts, and is particularly valuable in that it may equally apply to music-making in concert venues and to music-making for example in informal workshop settings. Thus, it offers a powerful foundation from which to bring the continuum of art for art’s sake through to art for social purpose into focus within HME. “Musicking” deconstructs dialectical opposition between the values of social interaction and the values of abstract art. Rather it creates shared ground between musicians’ artistry and social interaction, and opens up for widening perspectives on the potential of musical practises. This in turn highlights the importance of developing proactive approaches to curating “musicking” within communities and societies, encouraging emerging practitioners to sharpen their awareness of the creative, ethical and political dimensions of different practises, and to engage with the many choices they have, both in relation to expressing an artistic voice and in contributing to society as agents of positive change.

“Musicking” affords a turn toward action that may have particular societal motivations as well as remaining deeply connected to artistic values and practises. This is important in that it can embrace both more radical social and creative orientations in music-making (including participatory forms of performance that involve “audiences” in playing or singing alongside professionals, as well as diverse participatory workshop practises) and more conservative ones (including traditional concerts with audiences as listeners), and across the spectrum can point toward ways in which music constitutes something vital rather than simply decorative for people. The interdependence of imaginative, ephemeral, and social dimensions of music-making creates a compelling indicator of musicians’ potential to affect individuals and influence communities. As suggested by Soja (2010):

Human life is consequently and consequentially spatial, temporal, and social, simultaneously and interactively real and imagined. Our geographies, like our histories, take on material form as social relations become spatial but are also creatively represented in images, ideas, and imaginings… (Soja, 2010, p. 18)

Far from occupying a simple level of entertainment or enjoyment, music-making becomes a part of shaping individual and collective identities. This offers a compelling imperative for HME, and for example, as Hess suggests in focusing on young people: “The multi-faceted nature of musicking offers an important medium for youth to further develop their sense of justice” (Hess, 2019, p. 10).

### “Partnering Values” and Multiple Dimensions of Quality

With art for art’s sake and art for social purpose brought into dynamic dialogue grounded in “musicking,” a path opens toward the “partnering of values” (Campbell, 2016) for musicians and HMEIs, or in Lerman’s phrasing toward “hiking the horizontal” (Lerman, 2012), this being a manifesto against hierarchical thinking, allowing for different perspectives, co-existence of ideas, diverse spaces for performance, and a continuum of possibilities that demonstrate multiple forms of excellence. Both these concepts deconstruct dialectical opposition within professional practise, and rather look to embrace dialogical, reflective, and reflexive approaches, and forms of professional practise that respond directly to their context and communities with their creative potential.

**Figure 1:**
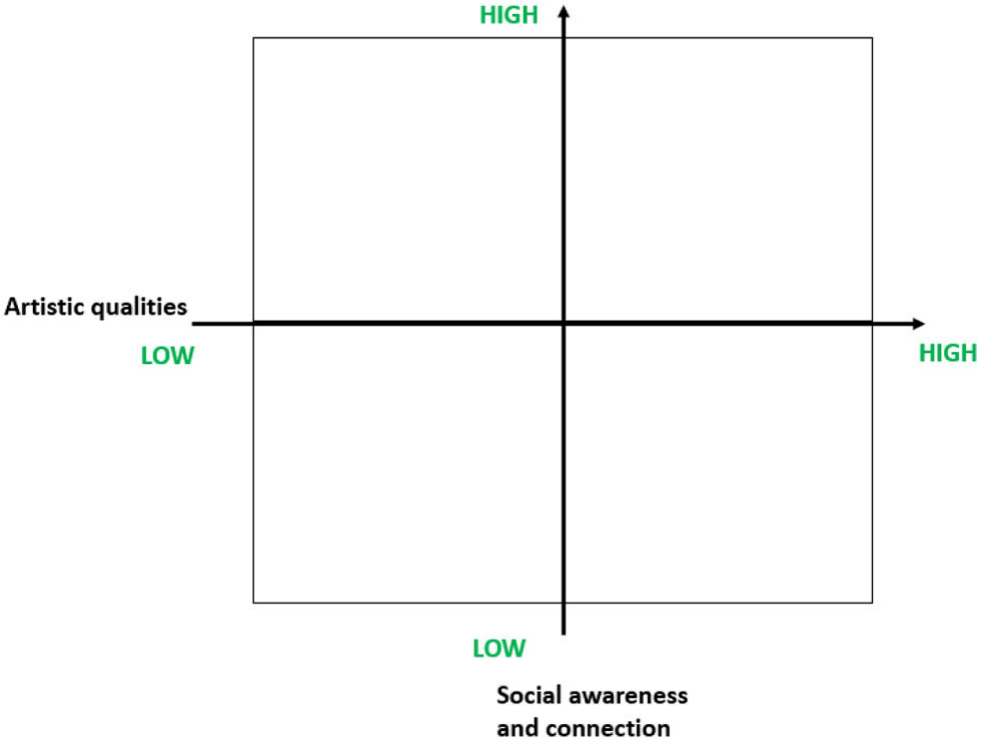
Combining artistic and social dimensions of quality in music-making.

This also raises critical questions about “excellence” and its measurement: how is “excellence” to be understood musically and/or socially? Alongside musical dimensions such as sound quality, technical skill and imagination, aspects of social awareness and interaction relating to “tolerance, generosity, [and] nimbleness” become critical (Lerman, 2012, p. 16). Dual axes of artistic and social concern are thus brought into dialogue with multiple possible outcomes in terms of excellence, as shown in Figure 1.

Criteria of achievement and what makes this excellent are of course visible within assessment frameworks used to measure the progress of students and to quality assure achievement of learning outcomes in HME (Jørgensen, 2009), but these may not always be as diverse and nuanced as the dual axes in Figure 1 suggest. Assessment criteria, together with the public profile that institutions choose to project, offer a powerful lens on an institution’s positioning of “excellence” and how this may combine focus on craft skills, individual musicianship, creativity, and artistry (the art for art’s sake end of the continuum) with practise in the real world, being proactively engaged with audiences and communities in society (social purpose). A traditional approach in many HMEIs has been publicly to celebrate success by lionising artists with exceptional international profiles as performers. This reflects a relatively narrow portion of the spectrum of “musicking” and one often associated with more traditional cultural structures of public concert halls/music venues. Few HMEIs, however, are likely to want to relinquish this label of “excellence” (Duffy, 2013), and indeed may perceive risks in expanding the continuum of possibilities.

Nevertheless, broader and contextualised criteria for excellence are proposed by Renshaw (2013, p. 51–53). He begins by articulating generic criteria for excellence that should be applicable to all forms of musical experience, whatever the context (for example, “focused listening to the music, to oneself and to the other musicians in the group” Renshaw, 2013, p. 51). He then considers specific criteria which, in the context of the western classical music of the HMEI, might start with “Mastery of the instrument, achieving a balance between technical and interpretative skill” (Renshaw, 2013, p. 52). He goes on, however, to indicate that for a music leader working in an informal education context, specific criteria for excellence and quality would be different. They would, for example, include “skill in managing and understanding the variables arising from the profile of the participants (e.g., age, numbers, experience, range of instruments, materials generated) and from the social and cultural context” (Renshaw, 2013, p. 52). Yet this does not mean consequently that “anything goes”; on the contrary it sharpens the need to understand measures of quality according to context and purpose.

It becomes increasingly important then for HME to reflect critically on “excellence” and how multiple dimensions of quality may be of value and assessed according to specific situation. Greater understanding of contextual dimensions for excellence is increasingly vital. Moreover, this will apply to all emerging musicians, whether they are looking to combine diverse forms of work in a portfolio career (as a performer, teacher, community musician, digital entrepreneur, or creative collaborator), or conversely be specialise in a highly focused career path.

### Critical Reflection and Reflexivity

With the need for critical reflection and reflexivity in HME already highlighted, questions arise about what they entail. The concept of the reflective practitioner (Schön, 1983, 1987) is well-established in music as in other professional disciplines, and combines processes of reflection-in and reflection-on action. Reflection-in-action takes place de facto as music professionals meet the messy realities of daily work; reflection-on-action is also likely to be embedded within the processes of maintaining and evolving practise.

Reflection is, however, gaining renewed attention within many professions including medicine and nursing, the law and teaching, as practitioners meet the unpredictable flux of contemporary societies (Billett and Smith, 2014; Cribb and Gewirtz, 2015; Dent et al., 2016). Equally, reflective practise is continuing to grow in higher education as part of supporting employability and sustainable practise (Barnett, 2012; Boud and Molloy, 2013; Coulson and Harvey, 2013). However, it has been more emergent and contested as an explicit part of HME (Alix et al., 2010; Tregear et al., 2016; Georgii-Hemming et al., 2020; Kruse-Weber and Hadji, 2020; Treacy and Gaunt, 2021), and even considered antithetical to practical learning and the *real work* of making art (Guillaumier, 2016). Part of the challenge here concerns the myriad possible forms of reflection, and the complexity of transcending purely descriptive reflection or reflection within a very narrow lens. Embracing forms of critical reflection that expand beyond the aim of improving musical craft skills, place artistic practise in context, and stimulate reflexivity (NAIP Strategic Partnership, 2017) is much needed in contemporary contexts. However, as these contexts create ever more intense pressures on time, expectations are increasingly to think “on the job” with little opportunity to step back and reflect critically.

Fundamental questions relating artistry to social responsibility and citizenship demand much more than thinking on the job. They require slow and reflexive work, with space for multiple perspectives to be voiced and considered. Development of reflective practise, therefore, that engages reflexively (both in-action and on-action) is beginning to be understood in music (Carey et al., 2017; Treacy and Gaunt, 2021; Westerlund and Gaunt, 2021) as in the arts more widely (Gielen and de Bruyne, 2009; Coles, 2012). This aligns with a conceptualisation that points beyond “effective” and “reflective” professional practise toward the importance in contemporary contexts of “enquiring” and “transformative” professionals (Gale and Molla, 2016; Carey and Coutts, 2021). “Enquiring” professionals are able to produce knowledge as well as implement existing expertise; are aware of the epistemological foundation of their practises and for improving these practises; and are also likely to belong to a community of similarly enquiring professionals, with whom they share the processes and results of their enquiry. Going a stage further, “transformative” professionals add a reflexive dimension to “reflective” and “enquiring” practises. This reflexivity involves practitioners’ critical reflection on themselves and on the social processes at the heart of their profession, and includes commitment to change and not just to understanding (Gale and Molla, 2016, p. 251–252).

Enquiring and transformative professionals both require time in which to deliberate away from the immediate demands of delivering work, not least as discomfort arises when existing beliefs are disrupted, and contradiction, inconsistencies and creative uncertainties have to be embraced. This is consonant with a perspective argued for some time that the contemporary pace of sociocultural change may ask HME to engage in *paradigm reflection* to review its fundamental purpose and goals and how these may evolve to better fit a changing contemporary world (Sloboda, 2011; Biesta, 2018). Such fundamental rethinking requires focused time and collective effort, critical reflection and reflexivity, but has potential to become “the stuff of high creativity, the setting of new trends, the reconceptualisation of the field, or the activity” (Sloboda, 2011, p. 13; Biesta, 2018).

## “Partnering Values” In Practise: Further Dynamic Relationships

The approach outlined above establishing a continuum between artistic and social purpose, and looking to a “partnering of values” has specific implications for several key aspects of HME. These include:

- Dynamic interaction both between established and less familiar repertoires and with the creation of new work;
- Relationships between imagination, artistic innovation and research, societal perspectives and experiences, and the cultural/social entrepreneurship required to make things happen in practise;
- Modes of learning, individually and collectively, including attention to the dynamics of power within learning environments and to the creative tensions between individual craft apprenticeship, ensemble work and collaborative inquiry.

These are addressed in the next sections. Here too, what may easily be perceived as conflicting or competing priorities are evident.

### Diverse Musical Traditions: Canon Repertoire *and* Making New Work

A central issue for HME concerns engaging with music as the preservation of cultural heritage and music as an art form creating new work. An important continuum extends between these. HME in the western classical tradition, for example, has a major presence at the heritage end of the spectrum, with a huge established canon of notated pieces by composers from different eras that has often formed the basis of its curriculum. Some performers may engage little with new work at the other end of the spectrum, and may not compose music themselves. A different position is held by popular musics where professional musicians are usually expected to be making new work at the core of their practise, paying less attention to reproducing existing repertoires. Specialising at either end of the spectrum may in itself be unproblematic. However, connexions across the spectrum, and some fluency with both established repertoire and creating new work, appear increasingly vital to practise in contemporary contexts because of the way they provoke musicians’ investigation of their own identities, their relationship to musical materials, and of the diverse social dimensions of “musicking” (Schippers, 2010; Avis, 2019; Bordin et al., in press). See also, for example, the direction of the Netherlands violin competition that places a particular emphasis on artists’ approach to “making” performance^3^ Such connexions illuminate possibilities in what it is and can be to “make” music, and suggest that combined artistic and social imagination offers potential to create hybrid approaches to the places and spaces of professional practises (Bhaba, 2004; Gielen and de Bruyne, 2009).

The teaching of core western classical repertoire (including for example some of the standard symphonic orchestral repertoire) has been central to most classical music programmes in HME, and may continue to be a priority in many contexts. Western classical represents music with long-standing traditions, and its canon repertoire has been accompanied by far-reaching implications for curriculum design, pedagogy, and indeed which students are recruited to HME. Teaching the next generations to keep this heritage alive at the highest levels has meant that pedagogy has consequently tended to be led by the practises and perspectives of master musicians within the tradition, with less focus on musicians’ own identities or vision. There has also been relatively little focus on the social nature of the “musicking” processes in relationships between performers and audiences or participants as opposed to relationships between musicians themselves. There is now, however, significant opportunity to rethink curriculum design and pedagogy for western classical music in the context of partnering artistic and social values, and with a “musicking” foundation. Furthermore, this approach looks promising as a way to support a flourishing future for a more traditional discipline within the increasingly diverse ecology of musics in society overall.

A number of approaches are already evident in this direction. For example, some HMEIs are increasingly embracing a more diverse range of repertoire and more diverse musical disciplines, with different canons and contemporary practises bringing alternative perspectives, curriculum structures and sometimes radically different pedagogies into the institution (Hill, 2009). This is opening up new possibilities for emerging musicians in terms of how they make work, with a variety of established and newer materials. How this is handled within an HMEI is clearly critical. On the one hand, respecting the integrity of different practises and the depth of their craft is vital. On the other hand, embracing diverse approaches may stimulate proactive curation of “musicking,” expand expertise, and may encourage musicians to engage more deeply with their own identities and vision, and with contemporary societal needs. This kind of approach to “musicking” starts to position musicians as “makers”—a term already familiar within some other art forms (Lees-Maffei and Sandino, 2004; Sweeny, 2017).

Ways in which repertoire is handled within other performing arts is also interesting to note. Theatre, for example, has been busy for a while transitioning toward a broader and more inclusive approach, including a growing practise of *de-centring* (McKenzie et al., 2010), which involves less focus on the canon of play texts. Overall there has been a clear move away from working with plays as artefacts in themselves to be reproduced perfectly and authentically, toward an approach that takes a text as a starting point for exploration and embraces the possibilities of more broadly configured types of performance (Kleiman and Duffy, 2019). Although this stance has been less prevalent within western classical music, it heralds creative possibilities.

Furthermore, the triangle of musician, music, audience offers a dynamic set of relationships to explore:

Live performance brings performer and listener into co-presence and establishes a relationship that works in both directions, even if it is highly asymmetrical […] (Cook, 2018, p. 18)

This issue of “co-presence,” which firmly positions the listener as an active agent within musical performance, offers a rich potential vein of development for HME, exploring forms of symmetry and asymmetry in the relationships between musician, music and audience. For western classical music, the emphasis in traditional concert settings has tended to be on the musician and the music, with the audience more of a bystander looking in on the action. This is a dynamic that as Kingsbury (1988) suggests may further serve to reinforce the notion of music-making as an observational rather than social encounter. There is, however, much potential to explore forms of greater symmetry in the relationships, as is increasingly encountered in live performance settings as diverse as musical theatre and rap and where it is more normal for the audience to “join in” (Diallo, 2019). It is not surprising then that fresh thinking and terminology for audiences (such as “experience seekers”) in western classical music are beginning to re-define audiences’ relationships with artists (Toelle and Sloboda, 2019).

Alongside what this may mean in the context of face-to-face, in person interactions, dimensions of “musicking” are also implicated in digital spheres. Thanks to the pervasiveness of digital and social media, some contemporary commentators are now using the term “communities” rather than the more asymmetrical “audience” to describe such multi-valent interactions (Partti, 2014). Belonging to a community is often proactively nurtured in such spaces—from singing along in a virtual rock choir to composing with collaborative virtual sound tools to posting a comment on YouTube classical music recordings (Waldron, 2018). A notable feature of these online communities is that the difference between amateur and professional is increasingly blurred, indicative of the “democratisation” of the internet. Furthermore, the dynamic, creative possibilities afforded by combinations of face-to-face and digital, live and asynchronous musical interaction, the world of games, virtual and hyper realities are increasingly being explored, and are dismantling “either/or” propositions such as one makes music acoustically or digitally but not both, or acoustic face-to-face music making is always preferable to digital making music (Waldron, 2018).

It seems critical, therefore, that HME engages with the concept of the “musician as a maker in society,” and with processes of “making” performances and “making” music with audiences and participants, whether these begin with canon repertoire or new work. This concept and principles are fundamental to contemporary music-making and to professional “musicking” across diverse musical disciplines. Furthermore, it seems vital that emerging musicians become aware of the co-curation and co-creation likely to be involved in their making processes, and that they are supported and able to navigate the resulting complexities of copyright and intellectual property that arise as more traditional boundaries of ownership blur.

### Artistic Imagination *and* Social/Cultural Entrepreneurship

Consonant with these perspectives, musicians who are “makers” are likely to find themselves navigating through a non-linear developmental journey, and one that particularly demands an enquiring attitude and ability to spot and embrace unforeseen opportunities. In this context, imagination (both artistically and in relation to society) needs to work alongside discipline and detailed research in developing ideas. Collaboration with others (quite possibly across professional disciplines) may also play a central part, along with producing and business acumen to realise things in practise and to find sustainable business models. An important continuum is therefore at play here, from imagination to innovation (Bridgstock, 2013; European Association of Conservatoires, 2014).

In recent decades research agendas in HME have been broadening and deepening, with a particular focus on research rooted in musical practises, including practise as research (European Association of Conservatoires, 2010; Borgdorff, 2012; Duffy and Broad, 2014; Skains, 2018). Furthermore, there has been considerable investigation of the interface and interconnections between research and reflective practise, connecting both with research through practise and ethnomusicological methods (NAIP Strategic Partnership, 2017; Treacy and Gaunt, 2021). A shared developmental core has been nurturing a research mindset in emerging musicians, which according to Sætre et al. (2019) means a mindset that champions curiosity and skills of exploration, and connects individuals more closely with their own artistic sensibility and voice.

A significant body of work has also looked at issues of employability over the last 30 years. This has brought important new dimensions to curricula, but has if anything tended to strengthen a sense of dichotomy between artistic and producing or business skills (Amussen et al., 2016). At this point in time, however, some of the most exciting potential for musicians lies in engaged imagination, and being able to bridge artistic mindset with societal need or interest, then facilitated by practical considerations and the business of making things happen (Gaunt, 2020). The question therefore of social as well as artistic imagination is important, bringing together advances in research and cultural entrepreneurship, connecting rigour, and structure to exploration, grounding artistic inquiry in contextual specificity and stimulating musicians to become active “makers” of their work in society. Conceived in this way, the continuum of imagination to innovation goes beyond issues of “employability” and getting work in a portfolio career (Bennett, 2016).

Connecting musicianship and musical creativity with social interaction and process, however, raises issues of empathy, social and cultural awareness, and fundamentally the need to embrace the social and ethical dimensions of artistic processes (Bazalgette, 2017; Wilson et al., 2017). In addition to responsibilities toward musical artefacts, social and ethical responsibilities have to be embraced relating to diverse forms of “co-presence” in music making in different settings. Improvisation practises provide powerful examples of this. As well as opening up new possibilities for musical exploration, divergent thinking and playfulness (in the sense of interactive experimentation, without necessarily having a fixed goal in mind), improvising entails social emergence, with concomitant elements of collaborative “not knowing” and a need to embrace failure as an inevitable part of sparking fresh expression (Sawyer, 2003; Clarke and Doffman, 2017). Embracing failure may feel diametrically opposed to the concept of “perfect” performance that can often be assumed to be the expectation. There is thus inherent tension to navigate in embracing failure *and* seeking excellent performance (Treacy and Gaunt, 2021). This is likely to be a feature across a diverse range of music, and certainly in improvised music, and requires careful contracting and ground rules for the interactions involved.

This becomes all the more pressing in the context of improvisation practises where these may be engaging diverse groups in society. The inextricable intertwining of creative and moral dimensions of improvisation has been evidenced by Smilde (2016), investigating professional musicians’ experiences of improvisation alongside group creativity of an improvisatory music practise with dementia sufferers. This work illuminates musical improvisation as a unique channel for making meaning and connecting people, and one that fundamentally explores the interdependence of artistic and social elements of “musicking.” It outlines the ways in which artistic and social process come together through improvisation, shaping and evolving identities both for the professional musicians and for participants living with dementia. Most importantly, this work also emphasises that improvisation is a practise that can [contra to some twentieth century notions that anything creative cannot be systematic or indeed systematically taught (Cook, 2018)] be nurtured in appropriately structured environments.

### Contemporary Apprenticeship: Individual *and* Ensemble

Enabling a shift from conflicting/competing priorities to a “partnering of values” within HME does not stop at the level of curriculum, artistic work, and entrepreneurship. A core set of issues concerns the detail of apprenticeship and pedagogies for HME.

There is plenty of evidence that the 1:1 lesson, a common signature pedagogy in HME, can and often does provide a creative and collaborative environment stretching both student and teacher, and resulting in transformational learning (Carey and Grant, 2016; Gaunt, 2017) where this concept is understood particularly in terms of learning that is both personally significant and enables individuals to make a shift from previous, more limited ways of being (Hodge, 2019). There is also evidence, however, that it may limit possibilities for student agency and creativity, particularly where teaching objectives focus narrowly on technique and prescribed repertoire (Jørgensen, 2000; Gaunt, 2010). Potentially toxic power dynamics of 1:1 tuition have been highlighted in some contexts in recent years (Dudt, 2012; Perkins, 2013; Oti Rakena et al., 2015; Bull, 2019). Much of this work has focused on western classical music, although the issues are not exclusive to this field, and a significant body of work is now emerging that emphasises the importance of more “student-centred” or “inclusive” pedagogies (Carey and Grant, 2016; Gies and Saetre, 2019; Gaunt et al., 2021). Furthermore, understanding is growing of the importance in transformational learning of real world experiences in diverse contexts in the field (Carey and Coutts, 2021).

Many HMEIs now aim to offer an increasingly mixed pedagogical menu, with 1:1’s complemented by diverse pedagogical approaches that are more socialised (to adopt Cook’s term) in terms of working proactively with the interpersonal and interactive learning potential of diverse group settings including peer groups; that foreground a research mindset (Duffy and Duesenberry, 2013); and that are more student-centred in terms of encouraging students to take responsibility for and shape the trajectory of their learning, individually and collectively (Hanken, 2016). In some contexts, resources are being invested to support artist-teachers’ pedagogical development (see for example Duffy, 2016). This expansion marks an opening up of apprenticeship, from a version narrowly focused on the transmission of practical craft skills to an artistic “making” process characterised by discovery and innovation, in which craft transmission is embedded. A single master-apprentice relationship may also become apprenticeship framed more within a community of practise enabling a variety of interactions and levels of engagement between people (Wenger, 1998; Kenny, 2016), and with individual expertise development sitting alongside ensemble development and the group creativity these may afford (Sawyer, 2006; Hakkarainen, 2013; Burnard, 2014; Gaunt and Treacy, 2019).

Expansion of one-to-one apprenticeship can usefully be framed by Jones’ conceptualisation of pedagogical stances, which outlines three types of relationships between teacher, student and subject content, with the teacher characterised in terms of a “gatekeeper,” “midwife,” or “fellow traveller” (Jones, 2005). The “gatekeeper” mode, perhaps most closely aligned with traditional apprenticeship, resonates particularly with an approach of transmitting knowledge from teacher to student, with the teacher making decisions about which material to offer when, and directing the student’s learning. The “midwife” mode has greater focus on the student determining their journey, constructing their learning process with varying levels of support and guidance from the teacher as appropriate. In “fellow traveler” mode, the most expansive and unpredictable of the three, teacher, and student embark on a shared journey, embracing learning on both sides and ready to explore unknown territories together.

The expansion of apprenticeship in HME, however, also often relies on an environment rich in ensemble work, one that includes interactions with a wide range of professionals, and where peer learning is championed alongside learning directed by a master teacher (Gaunt and Westerlund, 2013; Gaunt, 2017). Such learning environments are likely to promote engagement with personal choice, risk-taking and innovation, as well as helping to develop vital social elements of musical artistry (Dobson and Gaunt, 2015). Collaborative encounters through intercultural and international exchange may also open horizons, provoke critical reflection on fundamental values toward envisioning sustainable, just and ethical futures, and create space for students to consider their potential in contributing to societies (Gregersen-Hermans, 2020). Individuals’ understanding of the future contribution they can make through their own specialism, practices, and experience may thus be reframed (Fung, 2017). Facilitating such environments, however, clearly demands pedagogical agility, and highlights the importance of inclusive approaches and real world experiences underpinning the offering of professional expertise (Perkins, 2013). This again calls for fundamental rethinking in HME.

### Contemporary Apprenticeship: Group Creativity *and* Inclusive Pedagogies

The concept of group creativity (Sawyer, 2003), as well as being important to the pedagogy of apprenticeship, connects back to our earlier discussions about important relationships between artistry and the social dimensions of “musicking” in evolving professional musical practises. Cook (2018) aligns with this territory, examining how creativity develops from collaboration and social interaction rather than from the lone genius in “his” garret (although he stresses that the work of composers in particular who (still) often work alone should not be forgotten). Composition, of course, is already a popular discipline within HME, and, based on a making process is one which has much to offer contemporary contexts.

Cook’s perspective highlights the potential for multiple sources to inform expressivity and creativity for an artist; expressivity and creativity do not simply arise from the score, but may also emerge from the co-presence between performers (each with their own history of musical interests, skills, understanding, learning processes, and so on) and equally co-presence between performers and audience or community (whatever term is adopted). This perspective again shifts the concept of artistic citizenship from being one-directional and about an artist giving something back to society, to being about dialogical exchange where artists and their creativity are shaped by the experience, just as the identities and experiences of their participants may be. The significance of a dialogical understanding of “artistic citizenship” involving group creativity looks set to increase as firm cultural identities become more fluid within contemporary societies. In addition, recent evidence in particular indicates that an intercultural axis within group creativity may be transformational at multiple levels (Sklad et al., 2016; Ramnarine, 2017; Bartleet et al., 2020).

A further valuable dimension of group creativity concerns interdisciplinary collaboration. The recent trend for specialist higher arts institutions to combine into larger “Universities of the Arts” does not only concern financial considerations and aspirations to achieve economies of scale. Critically, it offers potential to facilitate forms of inter- and trans-disciplinary collaboration that underpin the kind of collective creativity and innovation that students will need to negotiate their multi-faceted professional careers (Alix et al., 2010; Renshaw, 2011; Ford and Sloboda, 2013). Many HMEIs are working to re-position their learning and teaching practises to promote collaboration (across artistic disciplines and/or for example with healthcare or tech disciplines) as an essential professional tool.

## Toward a Revised Conceptual Paradigm for HME

A refrain throughout this paper has been to urge HME to move away from a dialectic that tends toward competing or conflicting priorities to a conceptual lens that embraces a partnering of values and within this principle, greater attention to dynamic continua of possibilities as evidenced in section “Partnering Values” in Practice: Further Dynamic Relationships. Contemporary societal challenges create an imperative to evolve a conceptual paradigm for HME that reflects the interdependence of artistic and social dimensions of music making (highlighted in the concept of “musicking”), and brings artistic and social purpose into creative dialogue. A partnering of values from this perspective allows for more flexible and innovative, yet focused thinking about ways in which professional musical practises and HME can evolve for contemporary contexts, without throwing the proverbial “baby out with the bathwater.”

This makes a shift in the fundamental conception of HME and the roles that HMEIs may play within their local and global contexts. It is a shift that concerns musics and repertoires taught; forms of and contexts for music-making; who is engaged as audiences and/or participants; who gets to train as a professional musician; who works with student musicians and what pedagogies they use; and how practises of research, artistic endeavour, creative, and social entrepreneurship are integrated. All aspects of HMEIs are implicated: from buildings and physical spaces to organisational structures and development; from programmes, curriculum and pedagogies to student experience, public engagement, and stakeholder partnerships. Nevertheless, this is a shift that can also stay close to fundamental values in music-making.

**Figure 2:**
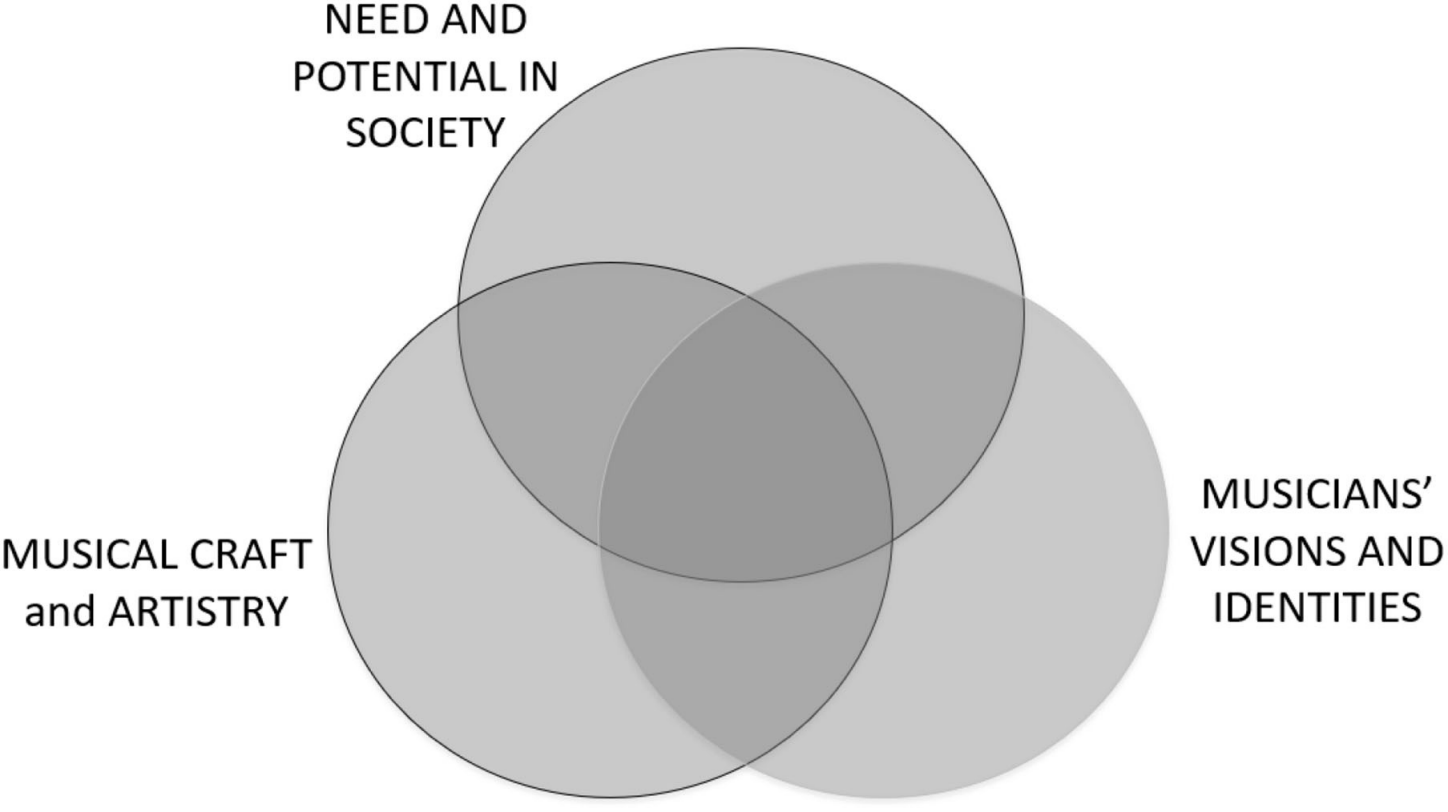
Three essential domains within a contemporary conceptual paradigm for HME.

We therefore propose a paradigm for HME that connects and champions the flow between a musician’s vision and identity on the one hand, and the practicum of society on the other hand (including the visions and identities of diverse people within society), with artistic craft and professional expertise as essential enablers. The paradigm brings these three domains together as shown in Figure 2. They are inextricably interdependent, each responding to and influencing the other, creating a finely balanced system.

Relationships between the three domains is further sharpened, for the perspective of an emerging musician in the twenty-first century, by drawing on Goleman’s concept of “triple focus” (Goleman, 2013). This looks toward a holistic and ecological frame of reference for professional practise in the context of hypercomplex, diverse, and fast-moving societies. In particular it addresses “attention” as a pressing contemporary issue in being of service and developing positive agency as a professional. Highlighting the problem of endless distractions invariably experienced from multiple directions, Goleman sets out the concept of “triple focus” as a series of three concentric circles, bringing self-awareness (inner circle), domain expertise (second circle), and being engaged with the big picture and horizon scanning (outer circle) into an interrelated whole. The application of this model within education has been highlighted (Goleman and Senge, 2016).

For HME the concept of triple focus re-emphasises the socially-embedded nature of “musicking,” connecting this both to social praxis (Renshaw, 2010; Elliott et al., 2016) and to the importance of artistic vision in society engaging musicians in their creativity/originality (Cook, 2018) as well as their socio-cultural and political orientations. Triple focus for HME embraces the imperative to look beyond music in abstract or aesthetic terms alone, to an ecology where the social dimensions of music-making (the relationships between musicians, musicians and their material, musicians and their participants including participants who may be called “audiences”) become interdependent. Goleman’s conceptualisation helps to dissolve polarisation between artistic and social domains, and to promote flow between artistic purpose, musical and professional expertise, and societal need/engagement.

### The Musician as “Maker in Society”

**Figure 3:**
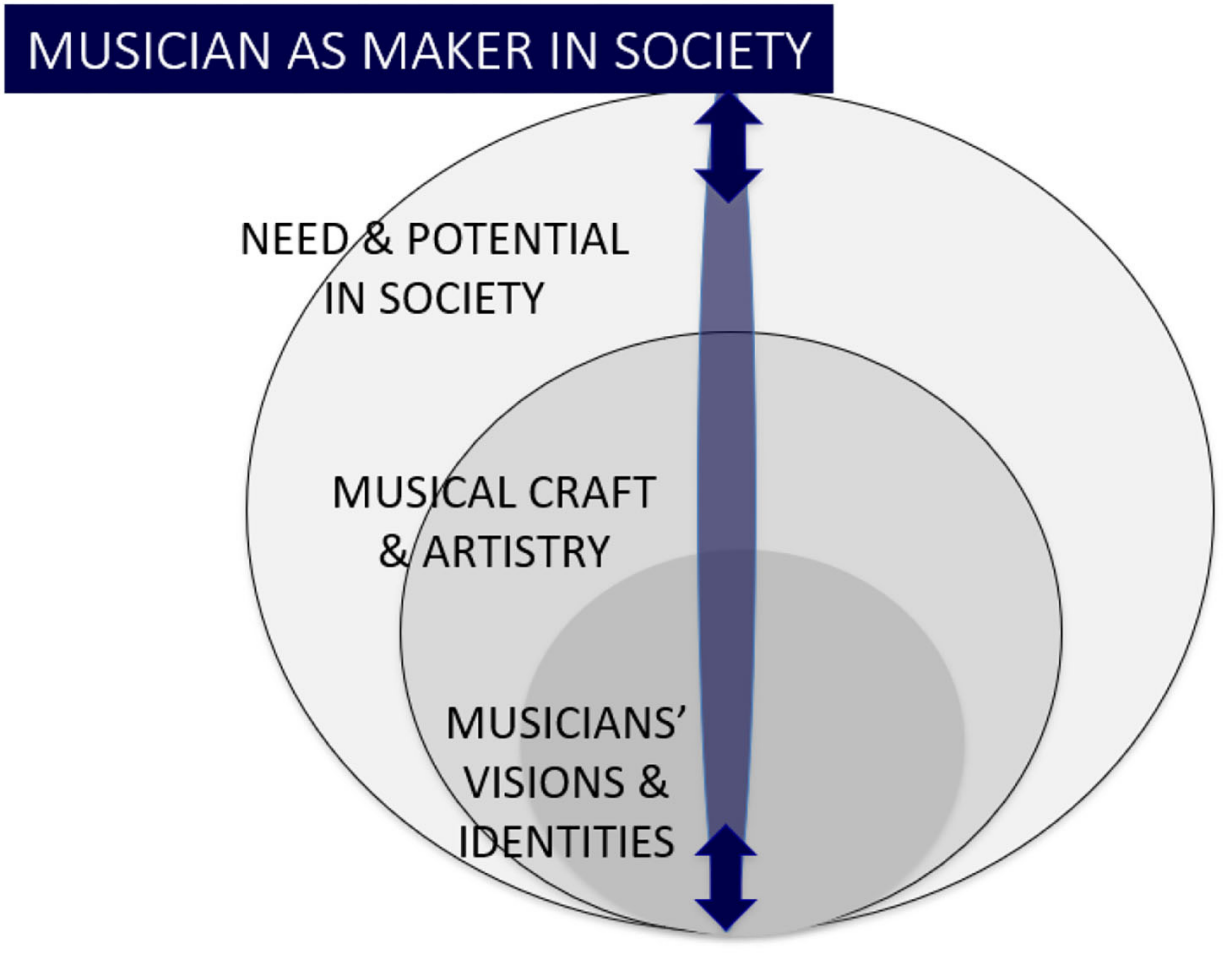
The musician as maker in society.

This in turn creates a foundation for professional practise that can be both artistically vibrant and connected to contemporary contexts, and where purpose for HME can be understood in terms of developing the “musician as a maker in society,” see Figure 3.

The musician as a “maker” retains a central value in craftsmanship (Sennett, 2009), its practical skills, embodied physicality and functional orientation. This attention to craftsmanship is particularly important in contemporary contexts where, as some have argued, ideas of creativity have been “elevated into an obligatory social order” that may not always be beneficial (Rechwitz, 2017, p. 5). Nevertheless, the musician as a “maker” highlights the importance of creative emergence involved in new work (see for example Hallam and Ingold, 2007). “Making” is distinguished from practises that simply “reproduce” existing work, something that may be attributed to playing canon notated repertoire, where musicians are afforded little creative ownership of their output (Rink et al., 2017). “Making” prioritises active interpretation and curating of performance and diverse musical practises in contemporary contexts. Equally, “making” recognises social orientation in musical craft, a process that is more expansive than being creative in aesthetic ways alone. The musician as a maker in society thus always creates afresh, *and* in relation to a specific situation. Context creates a unique situation and shapes possibilities for meaning to be made, whatever the materials used (repertoire or otherwise) and indeed within diverse processes of co-curation/co-creation. “Making” is not detached from the world. On the contrary, it embraces social situations, the spaces and environments of experiencing art, it embraces “musicking.”

In practise within HME, “making” opens up diverse ways for example into programming or engaging with audiences, or incorporating improvisatory dimensions as well as new composition into performance; equally it opens up for completely different ways of engaging with communities, collaborating or co-creating with them, evolving practises organically. The “musician as maker” foregrounds the importance of developing a relationship, individually and collectively as a community of practise, both with musical traditions and with the possibilities and demands of contemporary situations. In so doing the musician as “maker in society” also raises key questions about the motivation behind and objectives of musicians’ making processes and their impact. A shift in the preposition immediately signals the range of potential at play:

- Maker in society
- Maker for society
- Maker of society

Each concept here offers a different social, ethical and ultimately political orientation. Thus, the concept of the “musician as maker in society” makes a fundamental turn toward highlighting the inevitable moral and political roots to professional music practises. While these roots may not be explicit within many existing practises, the concepts of musician as maker in/for/of society start to raise awareness of their implicit relevance, and may be used to inform choices about the stance that individual musicians take and how these may shape professional practises.

### Evolving Curriculum and Pedagogy in HME

**Figure 4:**
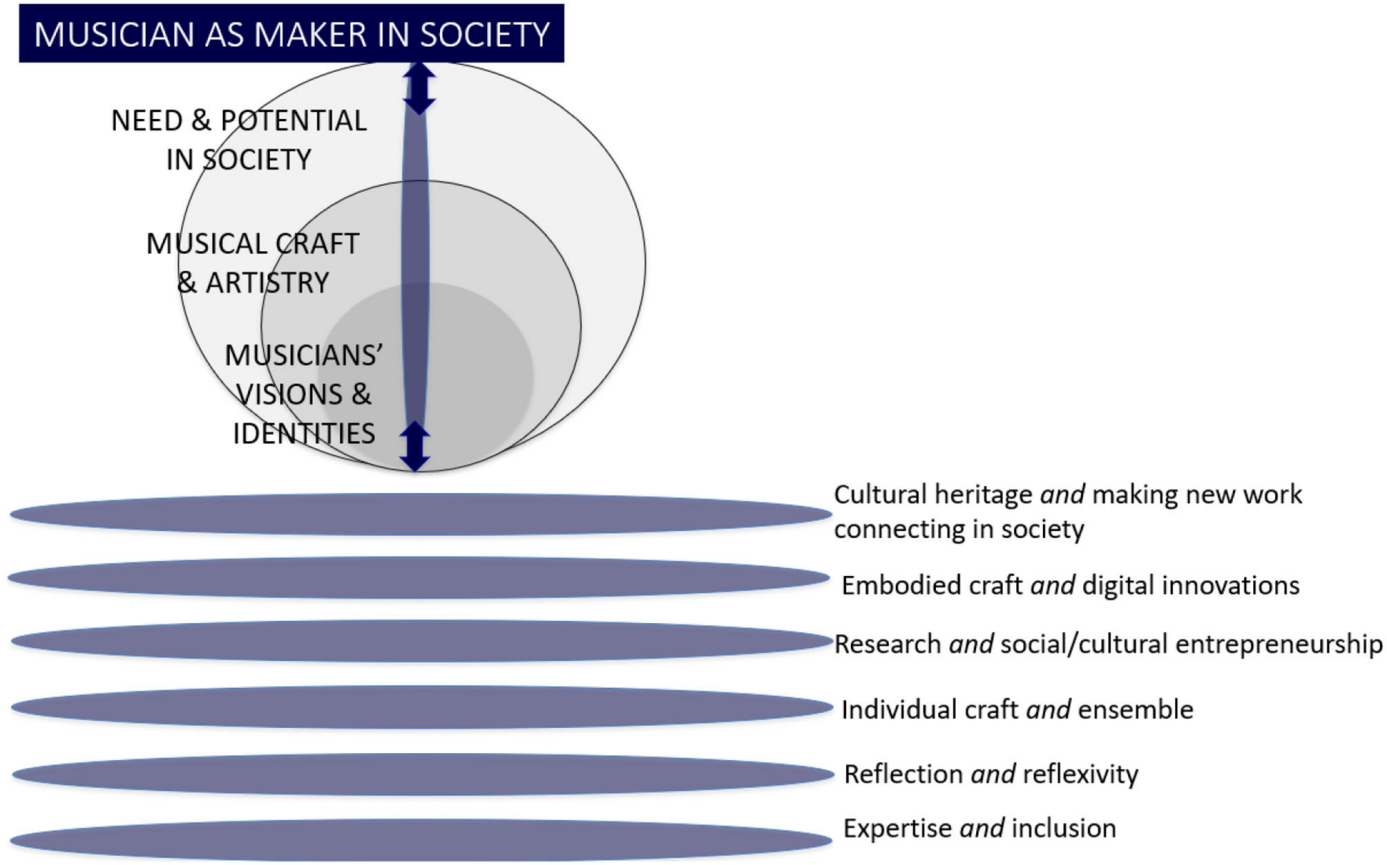
Mobilising lines of development for curriculum paradigm shift.

The musician as “maker in society” inevitably raises questions about implications for practise, and demands mobilising lines of development. Drawing on the major themes from our earlier discussion, we propose lines of development shown in Figure 4. In order that these should reflect the complexity of the discussion and further stimulate critical reflection on the importance of the fundamental principle of “partnering of values,” the lines of development are presented as dynamic continua each of which embraces creative tensions between elements within them that may be perceived to be competing or conflicting. In other words, the mobilising lines of development as dynamic continua aim to extend the dialogical approach to rethinking a conceptual foundation for HME argued for in this paper into the more practical level of determining specific curriculum activities and pedagogical tools, considering how they interrelate. Inevitably these dynamic continua are also interdependent, each having implications for the others. For example, the continuum “individual craft and group creativity” both makes significant demands on how “cultural heritage and making new work” may be shaped, and equally offers diverse and perhaps innovative possibilities.

#### Cultural Heritage and Making New Work Connecting in Society

The stance of “maker in society” is accompanied by a critical need to involve aspects of programming, improvisation, composition, and devising skills within core craft development for musicians from the beginning of their study, and in de-centring or de-colonising curriculum in ways that resonate with local contexts and contemporary societal issues. In addition, questions about assessment and how these reflect desired learning outcomes, and indeed inform ongoing creative development as an artist, are implicated. The notion of a “final recital” for example, presented within the bubble of an HMEI and for a few friends and family reflects a position that is distant from society—how might this be re-imagined as part of a more connected curriculum (Fung, 2017) and contemporary transition to professional life?

#### Embodied Craft and Digital Innovations

The embodied, face-to-face and live experience of music-making undoubtedly remains fundamental to HME when underpinned by the concept of “musicking.” At the same time, opportunities to engage with creative digital technologies are increasingly important to contemporary practises and interdisciplinary work: extending musical instrument capabilities and creating new instruments, devising immersive online and hybrid forms of performance, creating participation initiatives with particular communities, and finding ways to reach more diverse groups of people.

Embedding digital literacy skills, from recording techniques to fluency with communication platforms, forms of content streaming, and digital business models, is essential. Beyond this, opportunities to engage in multi-modal in-person and digital projects offer significant potential. With the digital world being so fast-moving, this is a field that is ripe for much creative research in HME, and for partnerships with the professional fields to create “laboratory” spaces as crucibles of skills development and innovation for professional practise.

#### Research and Social/Cultural Entrepreneurship

With the power of research through practise to connect students to their artistic identities, curiosity and creativity now well-established in HME, further work is needed into how research can support a stronger turn into society. Interdisciplinary questions relating to musical practises in society offer rich potential. In what ways may, for example, passions for research into historical performance practises connect with co-creating contemporary performance with diverse societal groups? Or in what ways may students research innovative forms of musical practise, for example working with those less able to access more traditional contexts for music: those living with dementia; young people excluded from school; people suffering depression or caught within the criminal justice system; early years children and their carers; older people experiencing isolation and loneliness, and so on?

#### Individual Craft and Ensemble

Individual practise and study as a musician are pre-requisite to professional success and enable profound engagement with music itself, but they do less to address critical aspects of the social foundations of music-making. The demands of significant solitary time if anything serve to highlight the vital importance of balancing individual work with ensemble experience, and of paying attention to the collaborative learning and group creativity these could nurture. Ensemble work begins to embrace both social and artistic dimensions of music-making. And it could create an important bridge to working further with the social dimensions, engaging creatively with audiences or participants in workshop settings, both in live situations and indeed through digital platforms. Ensembles and group work can stimulate group creativity, opportunities to engage across disciplines and to work in partnership with diverse organisations, and equally prompt shared critical and reflexive reflection that as we have seen has a vital contribution to sustainable professional practise.

#### Critical Reflection and Reflexivity

Critical reflection is particularly significant to the concerns of our conceptual framework in developing criticality within and about musical practises, their position in societies, and in supporting musicians’ reflexivity as a core part of their professional development. While reflection has often been associated quite narrowly with musical craft development, established career pathways, and with individual writing tasks, more diverse approaches that are also closer to artistic practise are now emerging in HME. These may include dynamic forms, such as interviewing visiting practitioners and analysing the material, or ensemble members reflecting together iteratively over time through practise as well as on practise, as they explore diverse forms of performance, interdisciplinary work, and forms of engagement in society. Such critical reflection may also usefully start to be made public in part, as a part of professional practise, for example through podcasts or blogposts.

#### Expertise and Inclusion

Maintaining respect for expertise within HME while opening the way to a more inclusive learning environment, and enabling greater student agency and responsibility, is a challenge. It demands sustained commitment alongside curriculum development to evolving pedagogy, with teachers engaging in ongoing critical reflection and professional development. This may include specific induction education for all students and academic staff; opportunities to extend skills and undertake training as the arts and higher education landscapes change; research opportunities and curated spaces for critical and collaborative reflection (including across disciplines); and interlinked pedagogical and artistic recognition for staff.

#### HMEIs as Resources of Hope, Imagination, and Innovation

As well-considering implications for the education of professional musicians in HME, the work of this paper also offers a provocation for HMEIs to reconsider how they themselves connect in societies in richer, more diverse ways, embracing civic mission, and knowledge exchange. HMEIs are increasingly extending beyond being institutions that train musicians for an established and predictable profession to take up a more dynamic position at the forefront of evolving the professions in contemporary societies. In other words HMEIs proactively innovate musical practises, engaging with local and wider socio-economic and environmental issues and their ethical and political dimensions, while remaining grounded in fundamental values of musical traditions. This marks a considerable turn, with HME undertaking greater leadership as an engine of renewal for the industries, and as a “resource of hope” (Williams, 1989) for contemporary contexts. This turn may indeed become one that is “spatial,” reflecting critically on the physical organisation of musical practises together with their complex socio-political dimensions and seeking to address issues of “spatial (in)-justice” (Soja, 2010). It is perhaps no surprise, and indeed a great opportunity that many students, with their growing voice in HME, seem particularly concerned to support such a turn, connecting to contemporary environmental, social, cultural, and political concerns, through and with their musical practises.

Such a mission for HMEIs has implications for how they become more accessible to their local communities. There are physical dimensions to this: how much doors are open; the visibility and audibility of activities from outside; or the ways in which activity connects with communities. Opportunities may include relaxed and accessible performances; free events that are informal and socially oriented; living room events in communities; community choirs, and ensembles with professionals and amateurs working side by side; an open curriculum offer without prerequisites for access; and partnership festivals in communities. Equally such a mission raises questions about connecting points with local issues, whether these concern mental health and well-being, stimulating creativity amongst business communities, or supporting disadvantaged groups from refugees to those caught in the criminal justice system. As well as partnering with big arts organisations (by now well-established in HME), there are key opportunities to partner with local charities, NGOs, social enterprises and community interest organisations both within the arts and other fields.

This kind of leadership for HMEIs offers potential to create laboratory spaces in which exploration, debate and innovation of musical practises in society can be catalysed, and sustainable initiatives can be developed through iterative stages. Such laboratory spaces lend themselves to being porous, enabling exchange between students, staff and visiting professionals, creating opportunities for interprofessional learning with other arts disciplines and across a wider range of professions such as medicine, nursing, architecture and urban planning.

Nevertheless, many HMEIs will have to undertake significant development to realise such leadership, and not least in terms of a strategic equality agenda to balance representation amongst both students and staff across all protected characteristics including race, disability, gender, and sexual orientation. Opening access and widening participation within student cohorts contributes a multi-layered and complex set of issues in itself. A range of initiatives and recruitment strategies engaged in diverse communities across specialist provision pathways under 18, short courses and summer schools are almost inevitably needed to support long-term change; and for music, unlike some other arts disciplines, particular emphasis has to be given to early years work where the long journey to professional expertise must so often begin. People and culture go hand in hand. Alongside reshaping recruitment processes, building greater flexibility into terms and conditions and connecting with diverse communities to establish networks that support individuals into working within HME, systematic attention to culture change may also be vital. This of course entails programmes of training and development, including unconscious bias and anti-racism training, inclusive pedagogies, coaching, and mentoring skills. Perhaps most of all it entails leadership development, starting from the top of organisations but critically recognising leadership as a core institutional practise that is distributed throughout the whole community (just as it applies to diverse musical ensembles). Proactively growing diverse leadership, particularly in early career professionals with HME has a significant part to play.

## Making Change

In this article we have been grappling with evolving purpose and practise of HMEIs in rapidly-changing societies. We have indicated multiple ways in which HMEIs have been developing in the last few decades, and have suggested that a paradigm shift is now needed to re-envision the conceptual foundation of HME. This paradigm shift rests on a social and moral turn based on embracing musical practises as social process (Small’s “musicking”) inextricably entwined with artistic concerns. For HME to look toward sustaining and strengthening professional music practises in societies, attention must be paid to this paradigm shift, and to the interdependent relationships it highlights between a musician’s vision, craft and artistry, and engagement in and for society.

We have proposed a conceptual foundation for HME that has potential to support this paradigm shift at institutional, curriculum and pedagogical levels, and we hope that it will also resonate with individual musicians. The “musician as a maker in society” is intended as a conceptual foundation for the twenty-first century that will help to focus appropriate change. It aims to nurture what Soja (2010) calls “strategic optimism”: critical thinking that embraces current realities (including their harsh dimensions), *and* unequivocally looks forwards to relevant and effective action. Such “strategic optimism” recognises and explores the potential of professional musical practises themselves to be resources of hope (Williams, 1989), “spaces” that fundamentally are open, invite imagination and may enable radical insight. For HMEIs such strategic optimism extends equally to the institution and its physical buildings. Our hope is therefore that the conceptual foundation proposed should enable the sector’s progressive evolution and growing value in societies. It may also be that some elements of its exposition will resonate across the performing arts.

There is little question that the path ahead for HME is as challenging as it is full of potential. With deliberation and transformative reflection being hallmarks of contemporary professionalism (Gale and Molla, 2016), it is clear that time and space are needed for rethinking the foundations for contemporary professional higher music education. Different institutions and contexts may call for more or less radical paradigm reflection (Sloboda, 2011) in doing this. The nuances are likely to be critical for example in resisting specific policy directions that have erred into polarised territory, looking to instrumentalise the arts and over-emphasise easily quantifiable measures of impact. A balanced view that puts artistic and educational values firmly alongside the demands of a professional marketplace is therefore vital. Maintaining a dynamic flow between artistic craft and imagination on the one hand, and societal relevance and engagement on the other hand, is a central and growing challenge. Furthermore, reflection and reflexivity seem increasingly essential to navigating the many pressing societal issues impacting HME as so many other sectors: diversity and inclusion, inequalities and discrimination, environmental sustainability, health and well-being, and profound disruptions of the digital revolution, to name but a few. Given the complexity of these issues, there is also a burning rationale for those in HME to reflect collectively. Networked deep thinking offers a powerful enabler of the group creativity currently needed. Within this, it seems particularly critical to amplify and deepen the place for student voices and the perspectives of emerging practitioners in different disciplines, with their concerns for the flourishing sustainability of societies, and their ability to carry the flame of professional music practises to the next millennium.

## Data Availability Statement

The original contributions generated for the study are included in the article/supplementary material, further inquiries can be directed to the corresponding author/s.

## Author Contributions

HG worked closely on initial material and then a first outline draft for the paper with CD. Following comments on this material from the remaining authors, HG developed the fundamental argumentation and detail of the paper, with an iterative process of further commenting from the other authors. All authors contributed to the article and approved the submitted version.

## Conflict of Interest

The authors declare that the research was conducted in the absence of any commercial or financial relationships that could be construed as a potential conflict of interest.

## Publisher’s Note

All claims expressed in this article are solely those of the authors and do not necessarily represent those of their affiliated organizations, or those of the publisher, the editors and the reviewers. Any product that may be evaluated in this article, or claim that may be made by its manufacturer, is not guaranteed or endorsed by the publisher.

## Footnotes

^1^ https://www.artsequal.fi/en/about

^2^ https://www.aec-music.eu/projects/current-projects/aec-sms

^3^ https://www.nederlandsvioolconcours.nl/english/
